# Ethics on academic procurement of cadavers

**DOI:** 10.6026/973206300200872

**Published:** 2024-08-31

**Authors:** KSV Angu Bala Ganesh, Payal Panda, Tanushree Gurawa, Prarthana Kalerammana Gopalakrishna, Saravanan Jagadeesan, Thirupathirao Vishnumukkala

**Affiliations:** 1 Department of Anatomy, Gujarat Adani Institute of Medical Sciences, Bhuj, Gujarat, India; 2 Department of Anatomy, C.U.Shah Medical college & Hospital, Surendranagar, Gujarat, India; 3 Physiology discipline, Human Biology division, School of Medicine, IMU University, Kuala Lumpur, Malaysia; 4 Department of Anatomy, School of Medicine, Lakeside Campus, Taylor's University, Selangor, Malaysia; 5 Anatomy discipline, Human Biology Division, School of Medicine, IMU University, Kuala Lumpur, Malaysia

**Keywords:** Ethical, cadavers, body donation, academics, research

## Abstract

Anatomy is a vital discipline in the realm of Medicine, and its primary means of study is through the use of cadavers. Experts in the medical, legal, and ethical domains have discussed the use of cadavers or their anatomical parts for educational purposes, which are considered a severe drawback. The advantages of using cadavers include their contribution to medical education, research, the investigation of innovative surgical procedures or techniques, the detection of anatomical differences at an individual or population level, the enhancement of surgical skills, and the support of other anatomical investigations. This review highlights the issues like consent, respect for the deceased and cultural beliefs on cadaver procurement practices and also it emphasizes the need for better body donation initiatives and public awareness campaigns to ensure sustainable cadaver procurement practices, ensuring consent, respect for the deceased and cultural beliefs.

## Background:

The utilization of cadavers in conventional medical education and training was initially introduced in Europe during the latter part of the Middle Ages. [1] Over the past 6-7 decades, the possibility of body donation has been considered as an alternate option, where individuals give their informed agreement to donate their bodies throughout their lifetime. [2] Currently, anatomical dissection is widely incorporated into medical education worldwide. [3] The use of cadavers in medical education is being increasingly replaced by alternatives such as manikins and virtual simulations of the human body. [4] The educational usefulness of these alternatives is still a subject of controversy. [5] Additionally, there is a growing trend towards the use of human cadavers in advanced medical education, leading to a continuous demand for cadavers in anatomical science departments across the globe. [1]

The process of obtaining cadavers for medical teaching and research relies on factors such as the level of awareness, willingness of the people, and local laws. [6] Currently, the primary sources of cadaver acquisition are from donating human bodies. [7] In 2012, the International Federation of Associations of Anatomists (IFAA) stopped ethically controversial procedures like acquiring unclaimed bodies. Instead, it promoted the donation of cadavers by individuals who have expressed their willingness to do so. [8] The utilization of cadavers without the consent of the deceased, particularly those from impoverished or mentally ill individuals, for anatomical studies has also faced criticism. [9] The IFAA rules were established to achieve global reach, however, there needs to be more information regarding the acquisition of cadavers on a global scale, and this issue may require further investigation in the future. [10] Only a small number of studies have discussed this topic, with their publications documenting the acquisition of cadavers from multiple countries across a single continent. [11, 12] This article provides a comprehensive analysis of contemporary practices concerning the procurement of cadavers for medical education on a global scale. We obtained this information through a comprehensive search of literature databases to investigate how cadavers are obtained for research and dissection. The purpose of this study is to determine whether the current procedures for procuring dead bodies can give rise to ethical concerns.

## Methodology:

The process of collecting global data was a significant challenge and led to the presence of inconsistent and uneven quality in the existing literature search. The literature search drew upon qualitative research (local surveys), quantitative studies (questionnaire studies with anatomists), and texts on medical education as pertinent information sources. The study thoroughly evaluated the current methods for obtaining cadavers for medical education and research worldwide, based on a comprehensive review of existing literature. The literature was searched using databases like MEDLINE, Embase, Google Scholar, Scopus, and Research Gate. A thorough literature search on ethical issues in cadaver procurement and anatomical actions was carried out using the snowball search method. This review encompassed the literature published within the last two decades. Over the past two decades, up-to-date information and comprehensive reporting from numerous countries have converged. In addition, our literature search revealed that there was a scarcity of published literature until the year 2000. The literature data on the sources of cadaver procurement in various nations was arranged by sorting and categorizing in a Microsoft Excel sheet (Microsoft Excel 2016, Version 15.30, Microsoft Corporation, Redmond, Washington).

## urrent practices of cadaver procurement:

Body donations and unclaimed bodies are significant contributors to the acquisition of cadavers worldwide, as previously mentioned. [3] Based on these two sources, the techniques for obtaining cadavers can be categorized as

[1] Exclusively relying on body donations

[2] Primarily, body donation is the primary source, with unclaimed bodies being a less frequent source.

[3] Primarily unclaimed corpses (and, occasionally, body donation)

[4] Solely unclaimed and may include bodies imported from other countries.

In this context, the term "donation" pertains to the assets or funds an individual bequeaths or contributes while alive. [13] Chile, Uruguay, and Venezuela obtain cadavers through body donations. [5] In North America, Canada, and the United States, bodies are donated, whereas Mexico and Nicaragua rely on bodies that have yet to be claimed. [14] European countries such as Austria, Czech Republic, Denmark, France, Germany, Ireland, Malta, Netherlands, Poland, Portugal, Spain, Sweden, Switzerland, and the United Kingdom acquire cadavers through body donation programs. [15] However, countries such as China, Japan, South Korea, Sri Lanka, and Taiwan acquire cadavers through body donation programs. [16, 17] South Africa, cadavers are acquired through body donation programs. [18, 19] Human tissues or organs used for anatomical research were mainly acquired as specimens from forensic departments during autopsies. [20, 21]

## Anatomy act for body donation:

The Anatomy Act is a regional legislation endorsed by the legislature and published in the official gazette of the state government. This legislation regulates the exploitation of deceased bodies for medical purposes; any individual has the right to donate their body for medical teaching and research. [22] There are no limitations based on age and health condition for being eligible as a donor, although the usage of the organ or tissue may differ. Individuals who test positive for specific screening tests may be rejected as donors due to potential risks to healthcare providers. [19] Many persons have become acquainted with and comprehended the prerequisites and have therefore contributed their bodies. [18]

## India:

The Anatomy Act was implemented in all states of India in 1948. This legislation allows individuals to give their bodies to medical institutes. [22] Presently, a significant proportion of the population in India willingly donates their bodies after death by completing a "pledge form" that requires the signatures of two witnesses. [23] The Anatomy Act of Punjab, enacted in 1963, authorized medical institutions and research facilities to utilize the bodies of deceased individuals. [24] To fulfil this objective, the Act advises the authorized official to acquire the corpse of a person who does not have a permanent address near their site of death or dies in public areas and is left unclaimed and delivers it to the authorities of an approved medical institution. [22] The Delhi Anatomy Act mandates relinquishing unclaimed bodies to medical institutes for anatomical teaching and study. It has provided guidelines to hospitals and public locations regarding donating unclaimed dead. [25] The Karnataka Anatomy Act of 1998 also provides a comparable provision for unclaimed bodies. [26]

## United States:

The American Association of Tissue Banks (AATB) accredits non-transplant tissue bank research and teaching programs to ensure that they fulfill or surpass the standards established by the AATB in terms of medical, technical, and administrative performance. Accreditation allows those who wish to donate their body to research or medical education programs to choose a program that has the highest level of quality. Non-transplant tissue banking and body donation is an exclusive industry that is subject to some level of control and does not pose any legal concerns. [27] Non - transplant tissue industries in the United States receive accreditation from the American Medical Education and Research Association (AMERA), a nationally recognized accrediting body. This accreditation applies to various entities, including bio-repository programs, tissue end users, university anatomical programs, and whole-body donor organizations. AMERA assists the industry in obtaining accreditation and actively participating in establishing standards suitable for non-clinical tissue industries. [28]

## United Kingdom:

The Human Tissue Act 2004 regulates the donation of human bodies in the United Kingdom, overseen by the Human Tissue Authority (HTA). This Authority grants licenses and conducts inspections of organizations, such as medical institutes, that provide education on anatomy using donated cadavers. In contrast to body donation laws in other countries, the UK Human Tissue Act permits individuals to donate their bodies for research purposes with their previous consent before death. However, it does not allow for the donation of a deceased person's corpse based on the consent of others. Additionally, an age restriction for body donation requires individuals to be at least 17 years old. [29] The Human Tissue Authority (HTA) functions are to provide donors with information regarding body donation and address several common inquiries regarding tissue donation. The HTA furnishes the affiliates with information about each organization, while each organization has its regulations regarding body donation. It also provides tools to help individuals identify contribution sites for those willing to donate. [30]

## Ethical considerations in the acquisition of deceased bodies:

This review aims to identify problems in obtaining cadavers in accordance with ethical norms. It will also examine the factors that influence the procurement of cadavers and ethical considerations related to religion and culture.

## Factors influencing the acquisition of deceased bodies:

There are multiple reasons for the decrease in the availability of cadavers for anatomical research and instruction. Some bodies may be denied for use in studying the normal anatomy of the human body because of the following factors: The examined cadaver, demise resulting from a transmissible or infectious ailment, decomposition, severe wasting, extraction of organs and tissue, and self-inflicted or homicidal death. The decision to accept or decline donated bodies is decided at the moment of donation. An institution has the prerogative to reject a bodily donation by regulations. [16]

## Cultural ethical concerns:

The views on body and organ donation as a benevolent act of contributing towards research, medical education and transplants are relevant. It suggests that the decision to donate is a personal choice. Individuals often need clarification on antiquated beliefs. Specific individuals may be reluctant to consent due to skepticism and conflicts of interest. [31, 32] One of the reasons for refraining from giving bodies was the concern caused by disrespectful behavior towards cadavers. The disposition of anatomists regarding donating their bodies for dissection remains largely obscure. [33, 34] The choice to become a body donor is affected by societal consciousness, cultural perspectives, knowledge of body donation, cultural perspectives and knowledge of mortality and understanding of the connection between the body and mind. [35] Research indicates that the primary motivation for most donors is their compassion for humanity and desire to contribute to the progress of medical education and posthumous assistance. [36] Another factor to consider is the contribution to assisting future generations and expressing appreciation for one's existence and well-being in the field of medicine in order to prevent a funeral or minimize wastage[37].

## Alternative applications for donated bodies:

Additional applications encompass scientific research and medical training. Enhance and develop novel medical technology; establish dedicated research partnerships in cancer research, Alzheimer's research, and advancements in surgical procedures. [38] Here are some instances of study that have been carried out using donated cadavers:

[1] Arthritis is the inflammation of one or more joints in the body.

[2] Cancer is a disease characterized by the uncontrolled growth and spread of abnormal cells.

[3] Cardiovascular Diseases

[4] Diabetes

[5] Knee/Hip/Shoulder Replacement

[6] Neurological Disorders

[7] Training for paramedics

[8] Management of spinal diseases

[9] Excision of Tumor

Some programs can distribute different body parts based on specific requirements, although only a few applications allow for the allocation of entire bodies. This ensures the maximum advantage from the donation.

Problems identified in the procedures of cadaver acquisition encompass.

[1] The majority of countries around the world rely on unidentified corpses.

[2] Only a few countries continue to rely on importing based on the laws. [5]

[3] Deceased individuals are still delivered to anatomists for dissection. [9]

[4] Several countries worldwide lack organized body donation programs.

[5] As indicated by several scholars body donation is mainly influenced by sociological and regulatory factors. [39, 40]

[6] Using anatomical structures disrupts the established ritual procedures. [9, 41, 42]

[7] The consent form presented to donors needs to include relevant information. [43]

[8] Variations in anatomical legislation across states or countries. [44]

## Strategies to promote a body donation program in a country:

In some countries public awareness campaign by local anatomists significantly increased the number of body donations. As a result, their department's main supply of cadavers changed from unclaimed bodies to donated bodies over a five-year span. [45] In Western societies, there was a significant shift in consciousness during the 20th century, leading to a greater acceptance of body donation. [1] The government and non-governmental organizations (NGOs) must actively promote and endorse the voluntary donation of bodies. Additionally, the public should be taught and fully aware of the importance of body donation to ensure an ample supply of human bodies in medical institutions. [46]

## Conclusion:

We examined publications that discuss different methods and ethical considerations related to the procurement of dead bodies, as mandated by anatomy acts in various states and countries. The study also identifies several ethical issues in body donation and procurement, outlined in these documents. These issues can serve as an agenda for addressing and resolving ethical concerns, aiming to regulate procurement methods and facilitate the supply of dead bodies for medical education.
